# Unexpected hemorrhage during robot-assisted laparoscopic prostatectomy: a case report

**DOI:** 10.1186/s13256-016-1030-4

**Published:** 2016-08-30

**Authors:** Shoko Nakano, Junko Nakahira, Toshiyuki Sawai, Noriko Kadono, Toshiaki Minami

**Affiliations:** Department of Anesthesiology, Osaka Medical College, 2-7 Daigaku-machi, Takatsuki, Osaka 569-8686 Japan

**Keywords:** Operative hemorrhage, Robot-assisted laparoscopic radical prostatectomy, Steep head-down tilt, Case report

## Abstract

**Background:**

Robot-assisted laparoscopic prostatectomy is increasingly performed as a minimally invasive option for patients with organ-confined prostate cancer. This technique offers several advantages over other surgical methods. However, concerns have been raised over the effects of the steep head-down tilt necessary during the procedure. We present a case in which head-down positioning and abdominal insufflation masked the signs of an intraoperative hemorrhage.

**Case presentation:**

A 73-year-old Asian man developed severe hypotension caused by an unexpected hemorrhage during robot-assisted laparoscopic prostatectomy for prostate cancer. Although our patient’s blood pressure steadily decreased during the procedure, his systolic blood pressure remained above 80 mmHg while he was tilted head downward at an angle of 28°. However, his blood pressure dropped immediately after he was returned to the horizontal position and abdominal insufflation – to create a pneumoperitoneum – was ceased at the end of surgery. We returned the patient to a head-down tilt to keep his blood pressure stable and began fluid infusion. Blood test results indicated that a hemorrhage was the cause of his hypotension. Open abdominal surgery was performed to stop the bleeding. The surgeons found blood pooling inside his abdomen from a longitudinal cut in a small arterial vessel in his abdominal wall, possibly a branch of his external iliac artery. The surgeons successfully controlled the hemorrhage and our patient was moved to our intensive care unit. Our patient recovered completely over the next few days, without any neurological deficits.

**Conclusions:**

We suspect that blood began to pool in our patient’s superior abdomen during surgery, and that increased intra-abdominal pressure suppressed the hemorrhage. When our patient was returned to the horizontal position and insufflation of his abdomen was discontinued, the resulting increased rate of hemorrhage caused a sudden drop in blood pressure. Surgeons and anesthesiologists must understand the hemodynamic changes that result from head-down patient positioning and abdominal insufflation.

## Background

Robot-assisted laparoscopic radical prostatectomy (RALP) is increasingly performed as a minimally invasive surgery option for organ-confined prostate cancer. RALP has various advantages over other surgical methods, including decreased blood loss and pain, shorter operation time and hospital stay, and lower complication rates [1]. However, concerns have been raised about the steep head-down tilt necessary during the procedure. This tilting can prevent recognition of deteriorating vital signs, causing complicated effects on patient hemodynamics, such as increased venous return or suppression of increased venous return during pneumoperitoneum. We report a case of delayed discovery of a hemorrhage caused by injury to a branch of the external iliac artery during RALP.

## Case presentation

A 73-year-old, 152.8-cm, 56.5-kg Asian man with a history of aortic valve insufficiency, hypertension, and postoperative deep vein thrombosis was scheduled for RALP to treat prostate cancer. His prostate-specific antigen concentration was 4.1 ng/mL. His prostate cancer had been diagnosed by biopsy 6 months earlier and his Gleason score was 4 + 3 = 7. He was undergoing complete androgen blockade therapy. Preoperative laboratory results showed a hemoglobin concentration of 13.5 g/dL, hematocrit of 38.8 %, and no abnormalities in his hemostatic function.

On the day of surgery, anesthesia was induced intravenously with 100 mg of propofol, 50 mg of rocuronium, and a constant-rate infusion of remifentanil at 0.3 μg/kg/min with inhaled desflurane at 5 %. We maintained anesthesia with inhaled desflurane at 5 % and intravenously administered remifentanil at 0.2 μg/kg/min in a fraction of inspired oxygen of 0.45. During surgery, our patient was positioned with a head-down tilt of 28°.

After almost 3 hours of surgery, at the surgical stage of urethrovesical anastomosis, our patient’s systolic blood pressure was <70 mmHg. At that moment, we administered 20 mg ephedrine, 300 μg phenylephrine, and 0.03 μg/kg/min norepinephrine to maintain adequate blood pressure. Arterial blood gas measurements showed a hemoglobin concentration of 10.2 g/dL and hematocrit of 30 %. We did not detect any problems with his respiratory parameters. Dissection of his pelvic lymph node was performed. The surgeons confirmed hemostasis.

The surgery was completed after ~4 hours, abdominal insufflation to create the pneumoperitoneum was ceased, and the operating table was returned to the horizontal position. Our patient’s blood pressure immediately dropped, with his systolic blood pressure decreasing to 40 mmHg. Repeat blood tests showed a hemoglobin concentration of 7.2 g/dL and hematocrit of 21 %. After administration of vasopressors, we placed a central venous catheter, with the patient in the head-down tilt position. We administered 120 μg of norepinephrine. After administration, our patient’s hemoglobin level fell to 5.9 g/dL, his hematocrit fell to 16.9 %, and his platelet count was 56 × 10^3^/dL. Because these findings indicated a possible hemorrhage, the surgeons immediately initiated open surgery. A large volume of blood and blood clots were present in his abdominal cavity and so we began rapid transfusion. His systolic blood pressure remained at 40 mmHg for 10 min; we performed chest compressions to maintain his blood pressure. The surgeons reduced the bleeding and maintained his blood pressure by manually pinching his common iliac artery and external iliac artery on his right side. They discovered a longitudinal slice in a branch of his right external iliac artery along the internal oblique muscle; ligation of this vessel achieved hemostasis. The branch was located around an instrument port that was located medially between a camera port and laterally to an assistant port.

**Fig. 1:**
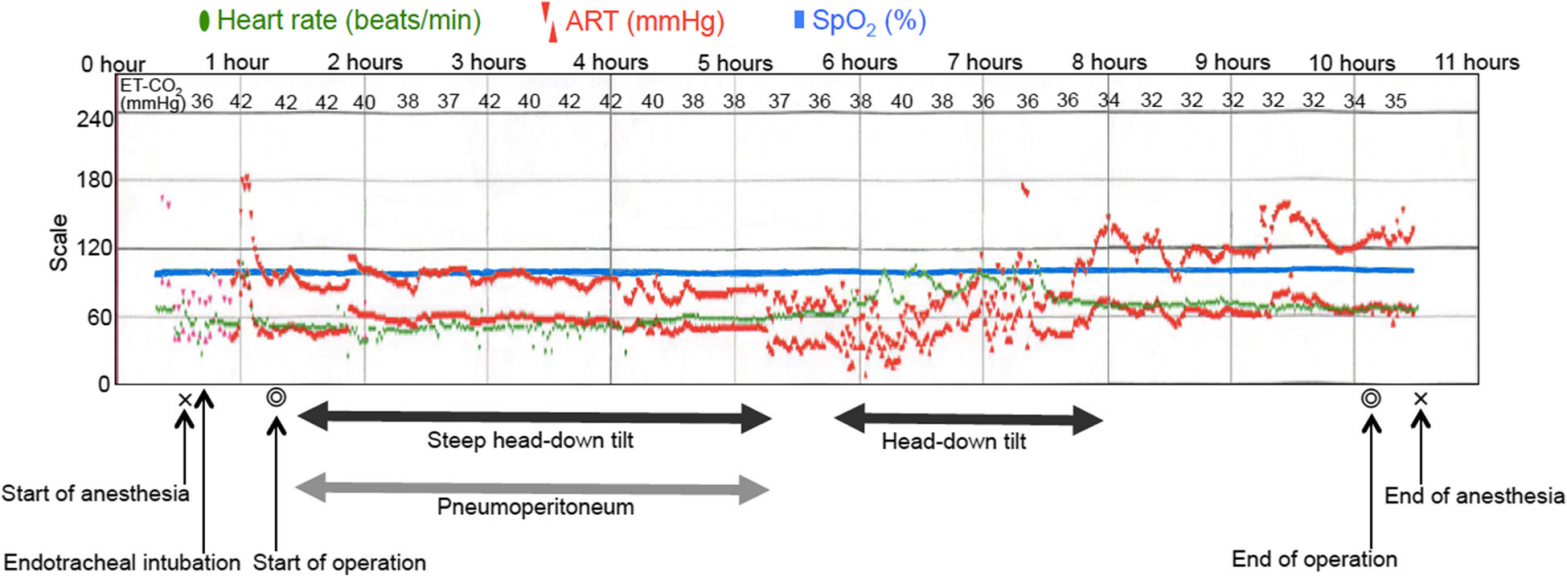
Anesthetic chart. *ART* arterial blood pressure, *ET-CO*_*2*_ end-tidal carbon dioxide, *SpO*_*2*_ percutaneous oxygen saturation

Surgery concluded after confirmation of hemostasis and blood pressure stability. The operation lasted 7 hours 50 minutes; anesthesia, 10 hours 14 minutes; and pneumoperitoneum, 3 hours 51 minutes. Our patient lost 5650 mL of blood and 350 mL of urine during surgery. Blood loss was estimated by measuring the blood in the suction bottle and gauze used to absorb the blood and blood clots. He received 5320 mL of red blood cells, 2400 mL of fresh frozen plasma, and 2500 mL of a 5 % albumin solution intraoperatively. His blood pressure, heart rate, and percutaneous oxygen saturation were recorded in the anesthetic chart (Fig. 1).

After surgery, our patient was admitted to the intensive care unit. The following day he was extubated and returned to the general care ward. He recovered from surgery with a suspected ileus and was discharged on day 29.

## Discussion

RALP is increasingly performed in patients with prostate cancer because it is a less invasive procedure than other prostatectomy methods. A previous study showed that RALP results in better surgical outcomes and fewer complications than laparoscopic radical retropubic prostatectomy [1]. Several postoperative complications, including pulmonary edema, central nervous system dysfunction caused by the steep head-down tilt (25–40°), and abdominal pneumoperitoneum with carbon dioxide, have been reported [2–5]. Anesthesiologists carefully avoid these complications during surgery and address postoperative complications promptly.

Many studies comparing RALP with retropubic radical prostatectomy have found that RALP reduces blood loss during surgery [3, 6–8]. Typical blood loss during RALP at our institution is less than 300 mL, including that passed through the urine. Patients generally do not need a transfusion, as many studies have reported. Our case was a rare occurrence of a hemorrhage resulting from an injury to a branch of the external iliac artery along the internal oblique muscle. We suspect that the vessel was transected during insertion of the laparoscope port at the beginning of surgery. The main reason the surgeon was unaware of the hemorrhage was the steep head-down tilt of the surgical table. We suspect that blood began to pool in our patient’s superior abdomen during surgery, and that the hemorrhage was suppressed by the increased intra-abdominal pressure. When our patient was returned to the horizontal position and gas insufflation was ceased, the resulting increased rate of hemorrhage caused a sudden drop in blood pressure. There was the possibility that an air embolism caused sudden hypotension when the table returned to its horizontal position, because the surgical site was at a higher level than the heart. However, our patient’s end-tidal carbon dioxide level did not change dynamically after the table was leveled. Anesthesiologists might have been able to recognize the gradual decrease in blood pressure caused by the hemorrhage. Although hemodynamics during RALP are complex, including increased venous return caused by the steep head-down tilt and restriction of this increase in venous return owing to pneumoperitoneum, two factors prevented us from recognizing the presence of a hemorrhage. First, the steep head-down tilt concealed the hemorrhage by increasing our patient’s blood pressure. Second, unrestricted fluid management concealed the hypovolemia caused by bleeding. Restrictive fluid management is generally recommended to prevent complications, such as pulmonary edema, laryngeal edema, and central nervous system dysfunction [9, 10]. However, restrictive fluid management is not performed in general in our institute at the surgeons’ request.

Anesthesiologists should be aware that approximately one-third of complications in laparoscopic surgery occur between the time of camera insertion and insertion of the ports [11]. The most frequent complication, occurring in 0.5 cases out of 1000, is injury to the retroperitoneal great vessels, including the abdominal aorta, inferior vena cava, common iliac artery, external iliac artery, and internal iliac artery. The second most frequent complication is intestinal injury, which occurs in 0.4 cases out of 1000. Injury to the abdominal wall blood vessels occurs less often [11, 12]. Additionally, several cases of bleeding from the port site have been reported [13, 14]. The abdominal wall blood vessels include the superficial epigastric artery and vein, superficial circumflex artery and vein, inferior epigastric artery, and deep circumflex iliac artery. Injuries to epigastric vessels, usually located between 4 and 8 cm from the midline and to vessels on the left side closer to the midline, have been reported previously [12]. Therefore, this area should be avoided as the entry point to the anterior abdominal wall. In most cases of injured epigastric vessels, bleeding at the port site is found as extraperitoneal hematoma or bleeding, and blood flows into the peritoneum through the defect created by the port when the port is removed. However, a hemorrhage in the present case was not found when the surgeon checked at the time of port removal. The hemorrhage was suspected only after the patient was returned to the horizontal position. Vital signs must be carefully monitored during and after alteration of patient position.

## Conclusions

In this case, a hemorrhage was caused by injury to a branch of the abdominal wall vessels during RALP. The increased intra-abdominal pressure resulting from pneumoperitoneum, the normal blood pressure resulting from a steep head-down tilt, and unrestricted fluid management prevented prompt recognition of the hemorrhage.

## Abbreviation

RALP: Robot-assisted laparoscopic prostatectomy
